# Estimation of the Craniectomy Surface Area by Using Postoperative Images

**DOI:** 10.1155/2018/5237693

**Published:** 2018-06-03

**Authors:** Meng-Yin Ho, Wei-Lung Tseng, Furen Xiao

**Affiliations:** ^1^Department of Neurosurgery, National Taiwan University Hospital, Taipei, Taiwan; ^2^Graduate Institute of Biomedical Electronics and Bioinformatics, National Taiwan University, Taipei, Taiwan; ^3^Department of Neurosurgery, Fu Jen Catholic University Hospital, New Taipei City, Taiwan

## Abstract

Decompressive craniectomy (DC) is a neurosurgical procedure performed to relieve the intracranial pressure engendered by brain swelling. However, no easy and accurate method exists for determining the craniectomy surface area. In this study, we implemented and compared three methods of estimating the craniectomy surface area for evaluating the decompressive effort. We collected 118 sets of preoperative and postoperative brain computed tomography images from patients who underwent craniectomy procedures between April 2009 and April 2011. The surface area associated with each craniectomy was estimated using the marching cube and quasi-Monte Carlo methods. The surface area was also estimated using a simple AC method, in which the area is calculated by multiplying the craniectomy length (*A*) by its height (*C*). The estimated surface area ranged from 9.46 to 205.32 cm^2^, with a median of 134.80 cm^2^. The root-mean-square deviation (RMSD) between the marching cube and quasi-Monte Carlo methods was 7.53 cm^2^. Furthermore, the RMSD was 14.45 cm^2^ between the marching cube and AC methods and 12.70 cm^2^ between the quasi-Monte Carlo and AC methods. Paired *t*-tests indicated no statistically significant difference between these methods. The marching cube and quasi-Monte Carlo methods yield similar results. The results calculated using the AC method are also clinically acceptable for estimating the DC surface area. Our results can facilitate additional studies on the association of decompressive effort with the effect of craniectomy.

## 1. Introduction

Decompressive craniectomy (DC) is a common neurosurgical procedure, and it involves removing part of the cranium, thus relieving an edematous brain and reducing intracranial pressure (ICP) by creating extra space. It is typically performed on patients with head injuries or stroke. Although the effectiveness of DC in controlling ICP has been demonstrated [1, 2], whether this control yields more favorable clinical outcomes remains controversial [2, 3].

A larger craniectomy might result in a more favorable ICP control [4], less delayed intracranial hematoma [5, 6], and more desirable clinical outcomes [6–8]; however, certain studies have reported that the craniectomy size is not correlated with complications [9], ICP control [9], or outcomes [10, 11].

The craniectomy size can be expressed in terms of the diameter [5, 8, 12–14], area [7, 9–13, 15, 16], or volume [17] of the skull flap. Although researchers in many studies have measured the craniectomy size according to its area, most of them have used a simplified formula to estimate the area. However, unlike the established ABC/2 formula for estimating the hematoma volume [18–20], the accuracy of such estimation of craniectomy area was never verified, probably because of the technical difficulties associated with computer-assisted area analysis.

In accordance with our previous study on the estimation of the skull defect volume [17], we implemented computer algorithms for surface area estimation and used them to validate a simple manual method of estimating the surface area of the skull flap by using postoperative images. To verify the robustness of these methods, data on small craniectomies, including posterior fossa and nondecompressive craniectomies, were also collected.

## 2. Methods

### 2.1. Patients

In this retrospective observational study, we collected 118 sets of preoperative and postoperative brain computed tomography (CT) images from patients who underwent craniectomies between April 2009 and April 2011 at the National Taiwan University Hospital. We included image sets of 75 male and 43 female patients. The indications for craniectomy were trauma in 50 patients, spontaneous cerebral hemorrhage in 29, cerebral infarct in 15, infection in 8, dural sinus thrombosis in 1, and brain swelling after various procedures in 15. Posterior fossa craniectomy was performed on 19 patients, and unilateral frontotemporoparietal craniectomy was conducted on the remaining patients.

### 2.2. Data Acquisition And Preprocessing

**Figure 1:**
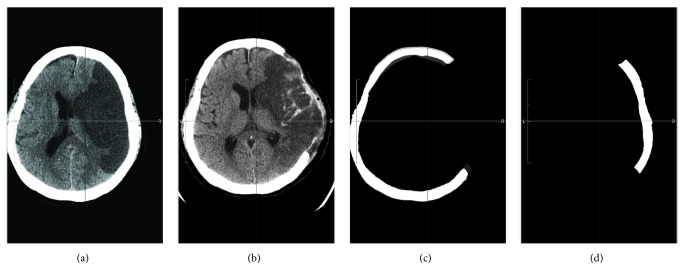
Illustration of image processing to generate the skull flap removed through craniectomy. From (a) to (d): preoperative images, postoperative images, registered postoperative images (bone only), and the missing skull flap by subtraction.

The preoperative and postoperative imaging procedures were performed according to a standard protocol of the hospital. All axial images were downloaded from the picture archiving and communication system (PACS) of the hospital to a personal computer for further processing. To obtain the craniectomy size, we first registered the postoperative image with the preoperative one. The missing skull flap was then generated by subtraction between the registered image pair. The processing is illustrated in Figure 1. Image segmentation and registration tasks were developed using C++ and the Insight Toolkit [21]. After the image analysis, the computed missing skull flap in each case was confirmed by board-certified neurosurgeons on a computer screen, slice by slice.

### 2.3. Surface Area Estimation

**Figure 2:**
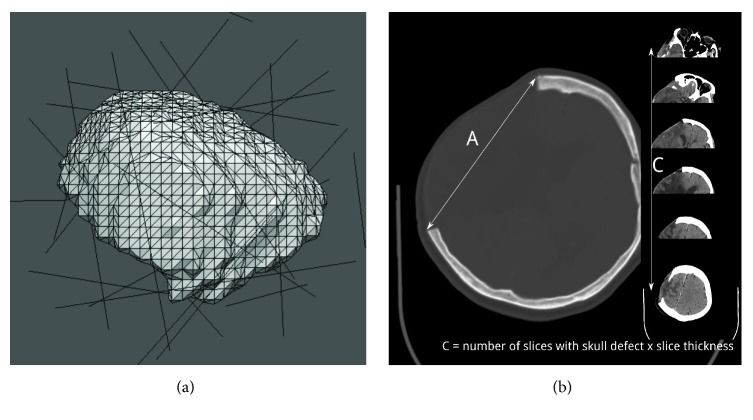
Illustration of the surface area estimation methods. (a) The marching cube method entails estimating the surface area by splitting the surface into small polygons (triangles in our implementation). The quasi-Monte Carlo method involves estimating the surface area by counting the number of intersections between the skull flap and lines generated using a low-discrepancy sequence. (b) AC method. *A* is the longest length of craniectomy on the axial slices, and C is the product of the number of axial cuts induced by craniectomy and slice thickness/spacing.

Three methods were used to estimate the surface area of the skull flap: marching cube, quasi-Monte Carlo, and our simple AC method (Figure 2).

The marching cube method is a computer graphics algorithm that creates polygonal models of constant-density surfaces from three-dimensional (3D) data [22]. We used the Visualization Toolkit [23] to create a triangulated representation of the missing skull flap and then summed the areas of small triangles. A skull flap mainly has two sides (inner and outer); therefore, we considered only triangles demonstrating a surface normal pointing outside the image center.

The quasi-Monte Carlo method is based on the Cauchy-Crofton formula from integral geometry. Liu et al. published the detailed methodology for applying this method for the surface area estimation of digitized 3D objects [24]. The method essentially entails estimating the surface area of a volumetric object by counting the number of intersection points between the object's boundary surface and a set of uniformly distributed lines generated using low-discrepancy sequences. Liu et al. further found that a desirable surface area estimate can be obtained using few thousand lines if a favorable low-discrepancy sequence is used. We followed this method to estimate the surface area of the missing skull flap. After generating a ball with a known surface area (*S*_*b*_) enclosing the skull flap, we used a four-dimensional Sobol sequence [25] implemented in the GNU Scientific Library [26] to generate 20000 lines in the ball. Furthermore, we counted the total intersection points between these lines and the skull flap (*n*). The skull flap surface could then be estimated by *S* = (*n*/*n*_*b*_)*S*_*b*_, where *n*_*b*_ is the number of intersection points between these lines and the enclosing ball, and its value was 20000 × 2 = 40000 in our implementation. The total surface area was then halved as the estimate of the outer surface area.

The proposed manual method can be termed the AC method, according to the ABC convention used to estimate intracerebral hemorrhage volumes [20]. The greatest dimension of the skull defect was identified after reviewing the axial brain CT images, and the linear distance between corners of the outer table of the skull defect was used to determine the craniectomy length (A) (Figure 2(b)). Furthermore, the craniectomy height (C) was determined by adding the interslice distance at which the full-thickness skull defect was visible on the postoperative CT images. The mathematical analysis involved in this method is highly similar to that reported in our previous study [17] and is included in the appendix because the mathematical part is subordinate to the theme of this study. In our study, one neurosurgeon estimated the surface areas of all postoperative images obtained using this method.

### 2.4. Data Analysis

After surface areas estimated through three methods were recorded, data were analyzed using R [27]. The Pearson correlation coefficient *r* was calculated using linear regression. We used the root-mean-square deviation (RMSD) to measure the pairwise differences in the surface areas obtained using two methods. Bland-Altman analysis [28] was also performed to assess the agreement between these methods. To conduct comparisons with previous estimation studies calculating the hematoma volume [18, 19], we also calculated the mean deviation (in percentage) between the various methods.

## 3. Results

Table 1 presents a summary of the surface areas estimated using the marching cube (Sm), quasi-Monte Carlo (Sq), and AC (Sac) methods. The estimated median surface areas were 134.52 cm^2^ for Sm, 131.73 cm^2^ for Sq, and 133.28 cm^2^ for Sac. Considering their mean as metaestimates, we determined that the craniectomy size ranged from 9.46 to 205.32 cm^2^.

**Figure 3:**
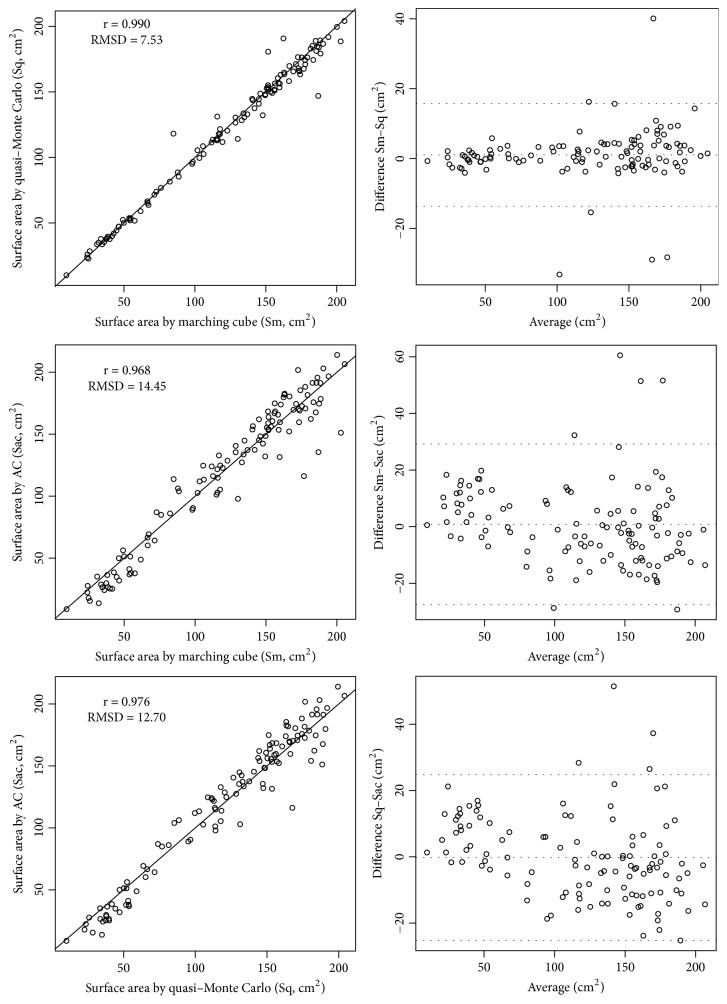
Data plots with the line of equality and Bland-Altman analyses between the craniectomy area estimated using the marching cube (Sm), quasi-Monte Carlo (Sq), and AC (Sac) methods. RMSD: root-mean-square deviation.

The data plots obtained using the line of equality and Bland-Altman analyses were our main results and are shown in Figure 3. Sm and Sq were similar, and their correlation coefficient was 0.990 and RMSD was 7.53 cm^2^. The mean deviation was 3.65%. Using Bland-Altman analysis, we determined that the 95% confidence interval (CI) for the bias was −13.66 to 15.69 cm^2^.

The differences between Sm and Sac were significantly higher than those between Sm and Sq. The correlation coefficient and RMSD were 0.968 and 14.45 cm^2^, respectively. In addition, the mean deviation was 12.12%, and the 95% CI for the bias was −25.57 to 29.20 cm^2^.

The differences between Sq and Sac were slightly smaller than those between Sm and Sac. The correlation coefficient and RMSD were 0.976 and 12.70 cm^2^, respectively. Moreover, the mean deviation was 11.41%, and the 95% CI for the bias was −25.18 to 24.79 cm^2^.

The paired *t*-test revealed that Sm and Sq demonstrated no statistically significant difference (*p* = 0.144). No statistically significant difference was observed between Sm and Sac (*p* = 0.543) or Sq and Sac (p = 0.865). In brief, the surface area measured using these three methods showed no statistically significant differences.

## 4. Discussion

DC is a neurosurgical procedure to relieve brain swelling. The craniectomy size might be correlated with ICP control [4], complications [5, 6], and clinical outcomes [6–8, 11]; therefore, the measurement or estimation of the craniectomy size is clinically relevant and should be routinely performed for every patient undergoing DC.

The simplest measurement of the craniectomy size is the skull flap diameter, estimated either during the operation or on postoperative images. Because of bone loss engendered by craniotomy and possible additional bone removal induced by the use of a rongeur, size estimation on postoperative images might be a more accurate approach, considering the advancement of PACSs. However, the simple anteroposterior diameter or largest diameter represents only one dimension of the craniectomy size. Therefore, measuring both the anteroposterior (AP) and superoinferior (SI) diameters is also common in clinical practice, although notations, such as 15 × 12 cm, complicate comparisons and statistics.

**Figure 4:**
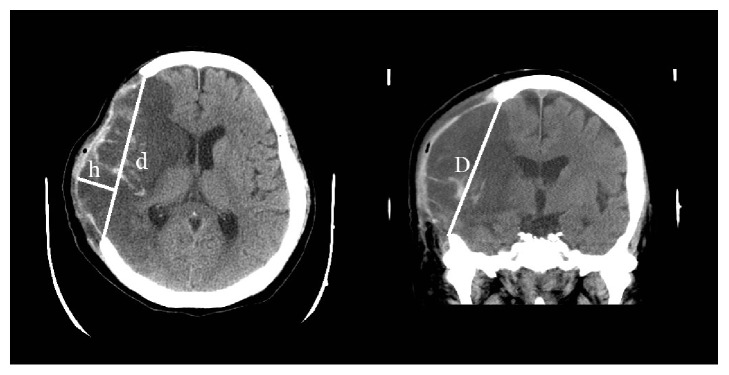
Previous attempts to measure the craniectomy area. The surface area can be estimated using the spherical cap formula: *A*_*s*_ = *π*[(*d*/2)^2^ + *h*^2^] [12, 13]. The base area can be estimated using *A*_*b*_ = (*π*/4)*d* × *D* [7].

The most common measurement of the craniectomy size is its area, either the base or surface area. If the craniectomy base is modeled as an ellipse (Figure 4), then its base area can be estimated by (*π*/4)*d* × *D*, where *d* and *D* represent the AP and SI diameters, respectively [7]. Although this estimation method is straightforward, validating it is difficult because the craniectomy base cannot be precisely defined to facilitate computer-assisted planimetry.

Studies have proposed estimating the craniectomy surface area by considering it as a spherical cap [12, 13] (Figure 4). The surface area of such a cap is *π*[(*d*/2)^2^ + *h*^2^], where *d* is the AP diameter and h is the longest distance from *d* to the dural flap. The formula ignores the SI dimension and estimates the protruding brain surface area instead of the craniectomy surface area; therefore, it is prone to change over the entire clinical course.

As per our review of the relevant literature, the present study is the first to validate the estimation formula through computerized methods. Our results revealed that the surface areas estimated using the marching cube and quasi-Monte Carlo methods were highly close because these two computerized methods are adequately established in the surface area estimation of digitized 3D objects. However, outliers exist, of which the difference may increase to 40 cm^2^. These images were typically degraded by metallic artifacts, which produced greater errors during the surface area estimation. We did not discard these data because we aimed to examine the robustness of these methods. Our results confirm the high level of agreement between these digital methods.

Even when the computerized methods were used, the estimated surface area of the digitized object is slightly different from that of the original object [24]. Our implementation of these two digital methods was also not perfect. In the marching cube method, we summed the area of small triangles exhibiting outward-pointing surface normals; this would inevitably include triangles on the edge in addition to the outer surface of craniectomy. Similarly, in the quasi-Monte Carlo method, dividing the total surface area by two may overestimate the surface area. This should not be a problem in large craniectomies but would contribute to inaccuracies in small craniectomies.

As expected, the agreement between the results of the AC method and those of the other two methods was not as high. Between Sm and Sac, the Pearson *r* value was 0.968 and mean deviation was 12.12%, and between Sq and Sac, *r* was 0.976 and the mean deviation was 11.41%. For comparison, a study reported that the correlation coefficient (*r*) between the ABC/2 method and volumetric analysis was 0.842 for subdural hematoma and 0.929 for intraparenchymal hematoma [18]. The mean deviation between the ABC/2 method and volumetric analysis was reported to be 14.54% [19]. Therefore, the effectiveness of the AC method in estimating the craniectomy surface area is similar to that of the ABC/2 method in estimating the hematoma volume, if not more favorable.

Certain factors render the AC method more useful than the digital methods. First, the AC method requires only the PACS for estimating the craniectomy surface area. Even if the CT image is placed in the traditional view box, we can still obtain an estimate from the film by using only axial slices. More accurate digital methods require extra processes, including segmentation and registration, which are typically not available on the commercial PACS.

The AC method requires only postoperative images to estimate the craniectomy surface area. By contrast, if preoperative images are unavailable for digital methods, we can only provide the mirror image of postoperative CT to estimate the surface area. However, the estimation may be error prone if the patient has an asymmetric skull or if pathology or artifact exists on the other side of the skull.

The AC method is the product of two length measurements; therefore, it should not be affected by most motion- and metal-related artifacts in images. Although digital methods are quite accurate, they might fail if severe image artifacts exist.

Our previous simple ABC method for estimating the skull flap volume is the product of the estimated surface area and bone thickness. However, the bone flap thickness might be erroneously measured because the thickness is usually the smallest dimension and is not even around the skull flap. For instance, we can expect a significant difference in the bone thickness measured between the bone and brain windows. We consider the AC method as a more robust method for estimating the craniectomy surface area; however, we also encourage investigators to record the skull flap volume estimated using the ABC method. When available, digital methods can be used to achieve a more accurate estimation of the craniectomy volume and surface area. If they are not readily available, we consider that the AC and ABC methods should be used to report the craniectomy size for associated studies. If only one measurement of the craniectomy size can be obtained, we recommend using the surface area estimated through the AC method, which is a simple, robust, and widely applicable method.

The association between the decompressive effort and craniectomy effect has been described in numerous previous studies. Studies have considered that DC may reduce the medical refractory ICP [29–32]. However, other studies conclude that the adverse effect of DC results in poor patient outcomes [13, 31]. Little evidence describing the necessity of craniectomy, appropriate timing, and clinical outcomes of different craniectomy sizes is available. Future studies regarding the immediate postcraniectomy effect, such as the change in ICP, reduction of the midline shift, or neurological improvement, can determine the correlation between the craniectomy size and treatment outcomes according to our approach. Studies may also evaluate the complication rate through various craniectomy sizes. They can also evaluate the ideal craniectomy size after further clinical investigation, and individualized preoperative plans can be devised in diverse circumstances.

The adequacy of craniectomy does not solely depend on its size. Other factors, including the extent of brain injury, space-occupying lesions, and systemic disorders, may also contribute to the effects of this procedure. However, these factors do not mean that a quantification tool for estimating the craniectomy size is not necessary or is not valuable. The methods we presented for estimating the craniectomy surface area provide tools for further investigation on the effects of size.

Our study had limitations. First, we examined the possible inaccuracy in small craniectomy procedures by using digital methods. Only one neurosurgeon evaluated all the craniectomy surface area by using the AC method; therefore, interrater agreement could not be established. Finally, this study focused on the craniectomy size; therefore, the effect of craniectomy and its association with size were not evaluated.

## 5. Conclusion

In this study, we compared three methods for estimating the craniectomy surface area. We confirmed that the marching cube and quasi-Monte Carlo methods were consistent. The accuracy of the simple AC method was also evaluated. These methods provide a quantitative evaluation for postoperative assessment in patients who have undergone craniectomy.

## Figures and Tables

**Figure 5:**
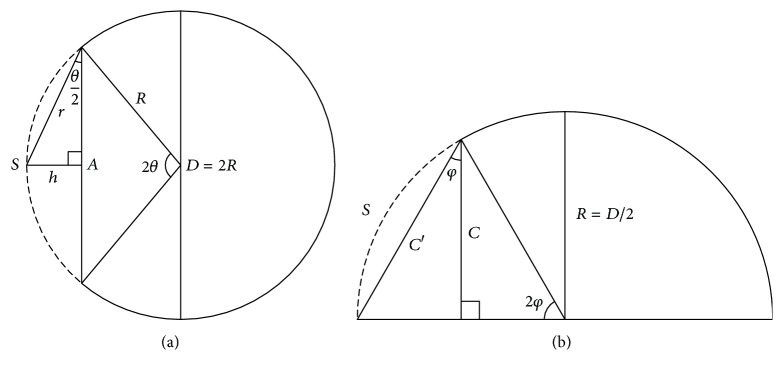
Schematic of the skull defect. (a) The skull is modeled as a spherical dome with the diameter* D* = 2R. The base diameter of the skull defect (*S*, dashed) is *A*. The apex angle of the spherical cap is 2*θ*. (b) Coronal view of the skull dome. The defect starts from the skull base, and the apex angle is 2*φ*. Although the height is* C*, the actual base diameter is *C*′.

**Table 1:** Surface area results obtained using different methods.

*Surface area(cm)* ^*2*^	*Marching cube(Sm)*	*Quasi-Monte Carlo (Sq)*	*AC method(Sac)*	*Mean ofSm, Sq, and Sac*
**Range**	**9.39 ~ 205.46**	**10.14 ~ 203.98**	**8.85 ~ 213.86**	**9.46 ~ 205.32**
**Median**	**134.52**	**131.73**	**133.28**	**134.80**
**Mean**	**120.58 ± 54.23**	**119.57 ± 53.28**	**119.77 ± 57.32**	**119.97 ± 54.54**

## Appendix

### Mathematical Analysis of the AC Method

The mathematical analysis of this method is highly similar to that reported in our previous study [17]. To simplify the derivation, we first model the convexity of the skull as a spherical dome with radius *R* and diameter *D*. We also assume that the craniectomy procedure is performed on a round piece of skull bone with diameter *A*. Therefore, the skull defect can be considered a spherical cap of the skull (Figure 5(a)). The apex angle, denoted as 2*θ*, is defined as follows:

(A.1) $$\begin{array}{c} \sin\theta =\frac{A/2}{R}=\frac{A}{D} \end{array}$$

An estimate of the surface area of the skull defect (S) is considered as that of the spherical cap with a base diameter of A [33]:

(A.2) $$\begin{array}{c} \hat{S}=2\pi Rh=2\pi R^{2}\left(\;1-\cos\theta \;\right) \end{array}$$

where *h* is the cap height. When (A.1) and (A.2) are used, the estimated surface area of the skull defect can be expressed as follows:

(A.3) S^=2πA2sin⁡θ21−cos⁡θ=π1−cos⁡θ2 sin2⁡ θA2=πA221+cos⁡θ=πA24 cos2⁡ θ/2

(A.4) S^=πA2cos⁡θ/22

The formula is similar to that of the area of a disk (*Area* = *πr*^2^), where *r* = *A*/2cos⁡(*θ*/2) in our case.

The skull is not a true sphere. If we model it as an ellipsoid, we can also obtain a similar estimate of *S*. However, the exact formula for the surface area of the assumed ellipsoid or spheroid cap is extremely complicated [34, 35] and beyond our need for a simple approximate formula. If we can simply obtain two orthogonal base axes of *A* and *A*′ of the skull flap, as an extremely intuitive generalization of (A.4), we can estimate the surface area of such a defect by using the following formula:

(A.5) $$\begin{array}{c} \hat{S}=\pi \left(\;\frac{A}{2\cos\left(\theta /2\right)}\;\right)\left(\;\frac{A^{\prime }}{2\cos\left(\theta^{\prime }/2\right)}\;\right) \end{array}$$

where sin⁡*θ*′ = *A*′/*D*.

Most craniotomy procedures are performed close to the skull base; therefore, we assume that the skull defect reaches the base of an imaginary dome and derive another axis from the coronal section (Figure 5(b)). The height (*C*) that we obtained from the axial slices of CT is shorter than the second axis (*C*′) of the ellipsoid cap that we aimed to investigate (Figure 5(b)).

(A.6) sin⁡2φ=CRC′=Ccos⁡φ

From (A.5), we know that

(A.7) S^=πA2cos⁡θ/2C′2cos⁡φ/2=πA2cos⁡θ/2C2cos⁡φ/2cos⁡φ=π4cos⁡θ/2cos⁡φ/2cos⁡φAC=βAC

where *β* = *π*/4cos⁡ (*θ*/2)cos⁡ (*φ*/2)cos⁡*φ*.

The value of *β* is dependent on *θ* and *φ*. It can also be expressed in terms of A/D and C/R. If both ratios are close to 0.8, as in most large craniotomy procedures, the factor *β* is close to 1 [17]. Even if the skull defect is extremely small or large, *β* rarely falls out of the range 0.8–1.2. Hence, we can assume that *β* ≈ 1 and estimate the surface area by using $\hat{S}=\beta AC\approx AC\equiv S_{ac}$.
